# Targeting autophagy process in center nervous trauma

**DOI:** 10.3389/fnins.2023.1128087

**Published:** 2023-03-06

**Authors:** Shanshan Wei, Bing Leng, Genquan Yan

**Affiliations:** ^1^Department of Pharmacy, Shandong Provincial Hospital Affiliated to Shandong First Medical University, Jinan, China; ^2^Department of Graduate, Shandong Academy of Medical Sciences, Shandong First Medical University, Jinan, China

**Keywords:** autophagy, central nervous trauma, spinal cord injury (SCI), traumatic brain injury (TBI), autophagy flux

## Abstract

The central nervous system (CNS) is the primary regulator of physiological activity, and when CNS is compromised, its physical functions are affected. Spinal cord injury (SCI) and traumatic brain injury (TBI) are common trauma in CNS that are difficult to recover from, with a higher global disability and mortality rate. Autophagy is familiar to almost all researchers due to its role in regulating the degradation and recycling of cellular defective or incorrect proteins and toxic components, maintaining body balance and regulating cell health and function. Emerging evidence suggests it has a broad and long-lasting impact on pathophysiological process such as oxidative stress, inflammation, apoptosis, and angiogenesis, involving the alteration of autophagy marker expression and function recovery. Changes in autophagy level are considered a potential therapeutic strategy and have shown promising results in preclinical studies for neuroprotection following traumatic brain injury. However, the relationship between upward or downward autophagy and functional recovery following SCI or TBI is debatable. This article reviews the regulation and role of autophagy in repairing CNS trauma and the intervention effects of autophagy-targeted therapeutic agents to find more and better treatment options for SCI and TBI patients.

## 1. Introduction

The central nervous system (CNS) functions as a data processor, coordinating and integrating various signals from the brain and spinal cord to regulate human behavioral activities (Reinhold and Rittner, 2017). Under the direct or indirect effect, CNS dysfunction can result in disorders of sensory, motor and nervous system functions, causing enormous physical and mental strain on patients and diminishing their quality of life (Hayta and Elden, 2018; Wang P. et al., 2018). In recent years, patients with Spinal cord injury (SCI) and traumatic brain injury (TBI) have shown accelerated development, bringing the issue into focus (Brown et al., 2012; Putatunda et al., 2018). Based on the mechanism of pathophysiology, there are two types of SCI: primary and secondary injury. The primary SCI is caused by external force factors, such as mechanical trauma or unintentional injury, which results in lesion region hemorrhage and dysfunction axon transmission. The secondary SCI is caused by primary spinal cord injury, and the residual normal nerve cells are permanently damaged due to ionic concentration imbalance, edema, and lipid peroxides overexpression (Anjum et al., 2020). The secondary injury process is long-lasting, more severe and affects a large area than the primary injury. Therefore, the primary focus of studying SCI is the treatment and mechanisms for secondary SCI that could limit the destruction range, identify neurological protection targets, and repair neurons in the spinal cord (Norenberg et al., 2004; Zhou et al., 2017; Wu et al., 2021). Clinically, spinal cord contusions are a common type of injury, accounting for approximately half of all SCI. TBI is comparable to SCI in that it can be divided into primary and secondary injuries. It can destroy the structure of the blood-brain barrier and the cellular environment through a series of cellular reactions, resulting in increased membrane permeability and exacerbated neurological system lesions (Hu and Tao, 2021). Secondary neural injury causes complex alterations and influences the pathway of autophagy after TBI. Specifically, brain damage increases the risk of neurodegenerative diseases in the elderly (Gardner and Yaffe, 2015). In addition, there is currently no universally accepted treatment for brain trauma.

The term “autophagy” refers to self-eating and dates back to 1963 (Ravikumar et al., 2010). Autophagy, triggered by external stimuli including hypoxia, inadequate nutritional intake or hyperthermia, is a conservative organism degradation and recycling process of clearing defective or incorrect proteins, toxic substances, and damaged organelles under the multiple lysosomal hydrolases (Levine and Kroemer, 2008; Glick et al., 2010). It keeps all eukaryotic cells at low activation levels and maintains cellular metabolic and homeostatic equilibrium (Parzych and Klionsky, 2014; Mo et al., 2019). When nutrition levels are adequate, autophagy can protect cells from abnormal proteins and damaged organelles, thereby preventing the development of certain diseases. Autophagy provides nutrition and energy for cell activities by degrading macromolecules and organelles during starvation. Blocking autophagy is the underlying cause of numerous diseases, including aging (Wong et al., 2020), infection (Deretic et al., 2013), cancer (Levy et al., 2017), and neurodegenerative disease (Guo F. et al., 2018). However, excessive autophagy can be detrimental to the organism, cause metabolic disorders and hasten cell death to an extreme degree (Maiuri et al., 2007). Three types of autophagy have been discussed thus far: macroautophagy, microautophagy and chaperone-mediated autophagy (CMA) (Glick et al., 2010; Mizushima and Komatsu, 2011; Parzych and Klionsky, 2014). Macroautophagy refers to the formation of an autophagosome with a double membrane structure to encapsulate intracellular material and the eventual fusion of the autophagosome with the lysosome. Microautophagy directly engulfs numerous cytoplasmic components by lysosomal membrane auto-transport (Zientara-Rytter and Subramani, 2016). It is the easiest way to disassemble the vesicle cargo. CMA contains an HSP70 complex that only identifies and expands KFERQ gene-encoded proteins and transports proteins into lysosomes by associated proteins on the lysosomal membrane surface (Kaushik and Cuervo, 2018). Generally, autophagy is the disorganized digestion of numerous components, including organelles with specific dysfunctions and long-lived proteins, including mitochondria and endoplasmic reticulum. CMA differs from others in that it is highly specific in mammals. In most cases, we are more concerned with macroautophagy, referred to as autophagy below (Mizushima and Komatsu, 2011; Parzych and Klionsky, 2014).

The review studies the fundamental mechanisms of autophagy and several common autophagy signaling pathways. We explore how autophagy affects the general two CNS insults and which drug has a protective effect against CNS damage.

## 2. Autophagy flux and related genes, autophagy signaling pathways

### 2.1. Autophagy flux and related genes

**FIGURE 1:**
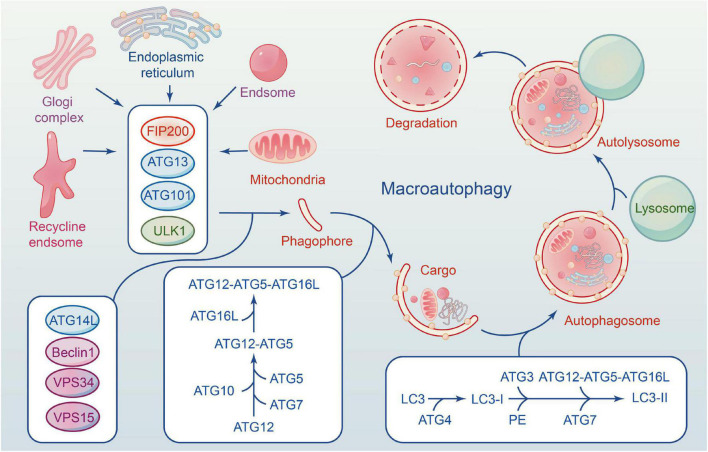
The formation of macroautophagy process and ATG proteins in mammalian cells. ULK1 complex involves in the induction of phagophores formation, and Beclin-1–Vps34 complex to enhances phagophores nucleation. Elongation of the autophagosome is mediated by two conjugated systems comprising ATG12– ATG5–ATG16L, and LC3–PE. After formation of complete autophagy vesicles, the mature autophagosome becomes fused with a lysosome to create an autolysosome, where sequestered molecules and organelles are degraded.

The autophagy flux reflects the dynamic process of autophagy-mediated cargo acquisition to degradation (Zhou et al., 2017; Wang P. et al., 2018). Phagophores are mostly formed by plasma membrane invagination, and their formation marks the beginning of autophagy. Accompanied by nucleation and expansion of phagophore until the formation of the autophagosome with a double-membrane vesicle structure that encloses various cytoplasmic cargo. Then autophagosome carrying cargo migrates toward the lysosome under the influence of the cytoskeleton, fuse to form the autolysosome and degrades their contents (Parzych and Klionsky, 2014; Zientara-Rytter and Subramani, 2016; Figure 1).

About 30 cores of autophagy-related genes (ATG) are involved in the whole process of autophagy flux (Ornatowski et al., 2020), and each gene has a distinct function at a specific stage (Levine and Kroemer, 2019). The ULK1 is a serine/threonine protein kinase in mammalian cells, and its deficiency disrupts the normal autophagy of cells (Zachari and Ganley, 2017). FIP200, ATG13 and ATG101 are essential proteins for phagophore induction; they interact with ULK1 and form a ULK1 complex (ULK1-ATG13-FIP200-ATG101). Activation of the ULK1 complex can induce autophagy in mammalian cells (Zachari and Ganley, 2017).

Beclin1 is phosphorylated by UKLI, facilitating the interaction of Beclin1, ATG14, VPS15, and VPS34 to form a PI3K III complex (Russell et al., 2013). The complex is necessary for autophagy’s nucleation. VPS34 is an essential component of the complex that phosphorylates the substrate phosphatidylinositol (PI) to induce the production of PI3P, one of the key components of the autophagosome membrane. Beclin1 is an ATG6 ortholog in yeast composed of an evolutionarily conserved domain, an N-terminal Bcl-2-homology-3(BH_3_) domain, and a central coiled-coil domain (Kang et al., 2011). Beclin1 binds with VPS34-15 after phosphorylating by ULK1. VPS34 complex (Beclin1-VPS34-15) selectively binds to distinct sections of autophagy, from formation to maturation, as determined by the types of binding proteins, including ATG14, Bcl-2, and UVRAG. The anti-apoptotic family proteins Bcl-2 can inhibit the formation of autophagosomes by interacting with Beclin1 through their BH_3_ domain. ATG14 interacts with the VPS34 complex to promote autophagosome elongation (Xu and Qin, 2019). UVRAG can mediate the fusion of autophagosomes and lysosomes. Therefore, Beclin1-VPS34 complex kinase activity is significantly correlated with autophagosomes, and Beclin1 can be used as an indicator of autophagy level.

Two ubiquitin-like complex protein systems affect autophagy, and both are advantageous to the extension or completion of phagophore double-membrane formation (Nakatogawa, 2013). The first is that ATG12 and ATG5 bind covalently under the ATG7 (E1) and ATG10 (E2) enzymes, forming an ATG12-5-16L multimeric *via* non-covalent bonding with ATG16L (Parzych and Klionsky, 2014). The second is ATG8/LC3 system (Schaaf et al., 2016). ATG8 is a homologous gene of microtubule-associated 1 protein light chain 3 (LC3) in yeast. ATG4 cleaves LC3 to form LC3-I during autophagy, and LC3-I binds with phosphatidylethanolamine (PE) to form LC3-II under the action of E1. ATG12-5-16L complex accelerates the fusion between LC3-II and membranes. The two systems are not completely independent and interact with each other. Numerous studies show that gene deficiency can result in autophagy defects, highlighting the significance of ATG genes in the entire process of autophagy flux (Glick et al., 2010). Kaizuka and Mizushima (2016) found that ATG13 knockout mice die *in utero*, and their fibroblasts are incapable of forming autophagosomes. Another piece of literature is consistent with this point *via* inactivating ATG5 (Huang et al., 2021).

We typically measure the level of cellular autophagy using biomarkers in a western blot or immunofluorescence experiment, as the intensity of autophagy flux varies in response to external stimuli. Evidence documented that increasing autophagy in mammalian (Lai et al., 2008) and cellular experiments (Wang et al., 2014) of SCI or TBI by alteration in biomarkers persisted for at least 1 month. Although this method is simple, rapid and quantitative, each marker has its limitations that must be thoroughly considered when selecting markers. The following key proteins have been tested for autophagy in recent years: LC3 (microtubule-associated protein 1 light chain 3), p62/sequestosome-1(SQSTM1) (Zhou et al., 2017).

Light chain 3, an ATG8 ortholog in yeast, is the universal marker that participates in autophagy membrane formation. There are two forms: LC3-I and LC3-II, that can be transformed into one another. In autophagy, when LC3 protein is synthesized, ATG4 immediately cleaves at its carboxyl-terminal Gly120 resulting in the formation of LC3-I. The ATG12-5-16L complex can regulate the ATG3 activity. They work with E1 closely to promote the combination of LC3-I and PE, thereby establishing the relationship between LC3 and autophagic vesicles (Tanida et al., 2008). Consequently, LC3-II or LC3-II/I are frequently used to describe indicators of autophagy flux alterations. It has been suggested that autophagy is significantly activated by the amounts of LC3-positive cells and autophagy vacuoles from 4 h to 21 days in the lesion site of SCI (Kanno et al., 2011).

P62, also known as SQSTM1, is an adaptor protein with multiple regions: N-terminal Phox1 and Bem1p region, the Keap1-interacting region, the ubiquitin-associated protein region and the LC3 binding region. It is primarily responsible for degrading unwanted proteins and damaged cell components. After autophagy initiation, P62 binds to ubiquitinated proteins and forms a complex with LC3-II, which is located on the autophagosome endomembrane, then phagophore envelops complex enters into the lysosome for degradation to achieve recycling. The P62 protein levels are negatively correlated with autophagic flux activity. Consequently, the degradation of P62 is typically used as a variable in autophagy level analysis (Pankiv et al., 2007; Lamark et al., 2017).

### 2.2. Autophagy signaling pathways

The mammalian target of rapamycin (mTOR), a key cellular regulatory factor, coordinates nutrition, growth and metabolism. mTOR interacts with RAPTOR, PRAS40, mLST8, and DEPTOR to form a mTOR complex 1 (mTORC1) (Wang and Zhang, 2019). mTORC1 is only activated when the cell has growth factors, adequate glucose levels, nutrition and sufficient oxygen; otherwise, it is inactive. The level of cellular nutrition is proportional to mTORC1 activity. The mTORC1 acts as a negative regulator of the actor on the ULK1 complex of autophagy regulation. Under adequate nutrition, mTORC1 can bind to the Ser757 on ULK1 to inhibit the formation of the ULK1 complex and achieve the effect of inhibiting autophagy. In contrast, its activity decreases under energy starvation conditions or in rapamycin (TOR) presence. Then ULK1 complex is activated and phosphorylates ATG13 and FIP200 to induce autophagy (Kim et al., 2011). The purpose is to maintain the nutrition required for cell survival. After CNS injury, inhibiting the mTOR pathway significantly impacts autophagy, according to numerous studies (Guo Y. et al., 2018).

**FIGURE 2:**
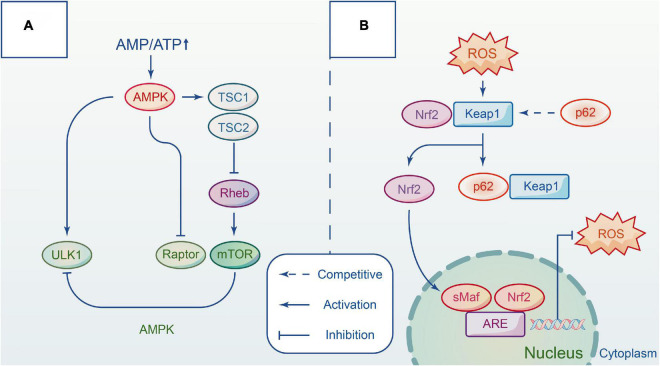
The main pathways involved in autophagy by interacting with different complexes. **(A)** Autophagy is regulated by AMPK and mTOR signaling pathway in different ways. Upon the energy depletion, AMPK responds to energy changes, and repress mTORC1 to modulate ULK1 activity and induce autophagy. **(B)** Keap1-Nrf2 signal pathway is a defense mechanism to protect the body from oxidative stress. The adaptor protein p62 competitives with NRF2 and forms a p62-KEAP1 complex. Hence, Nrf2 accumulates in the cytoplasm and then translocates to the nucleus to initiate transcription, which provides an antioxidant action. Repress mTORC1 to modulate ULK1 activity and induce autophagy.

Adenylate-activated protein kinase (AMPK) is a highly conserved serine/threonine protein kinase that acts as an energy change sensor to maintain the energy balance between catabolism and anabolism in cells. It comprises three subunits: α catalytic subunit, β and γ regulatory subunits. Multiple studies have shown that AMPK activity is affected by the intracellular ratio of AMP/ATP (Lin and Hardie, 2018). Under normal conditions, AMPK is inactivated, and the cell has minimal AMP. When energy is insufficient, AMPK is activated. It induces autophagy progress in three ways, directly or indirectly acting on the ULK1 complex (Kim et al., 2011). TSC (tuberous sclerosis complex) is an upstream negative regulator of mTORC1 with GTP-activating enzyme activity. Rheb (Ras Homolog Enriched in Brain) is a GTP enzyme that positively regulates the activity of mTORC1 by combining GTP (Pereira et al., 2017). AMPK inhibits the growth of mTORC1 by combining TSC with Rheb after phosphorylation. AMPK directly inhibits mTORC1 activation by competing with the substrate for mTORC1 RAPTOR, thereby reducing substrate recruitment. In addition, AMPK interacts directly with certain sites on ULK1 to regulate autophagy, such as Ser467, Ser317, and Ser777. Other evidence indicates that AMPK also affects Beclin1 S91/S94 sites to upregulate autophagy (Kim et al., 2013), but further study is required. It has been reported that activating the mTOR and AMPK signaling pathways can induce autophagy to protect against lysosomal damage (Jia et al., 2019; Figure 2A).

The principal cascade signaling pathway that regulates mTORC1 is the PI3K pathway, which can be divided into three classes. The increase in Complex III is caused by the activation of PI3KIII, resulting in autophagy activation. However, it generates PI3P after classIPI3K is activated, as stated previously. PI3P acts as a second messenger that regulates the downstream PDK1 protein and activates AKT protein, thereby inhibiting TSC, activating mTORC1, and finally mediating autophagy (Carloni et al., 2010; Ersahin et al., 2015). Activating the PI3K/AKT signaling pathway is beneficial for nervous system disorders (He et al., 2022).

Kelch-like ECH-associated protein 1 (Keap1)-nuclear factor-erythroid 2-related factor 2 (Nrf2) signaling pathway is considered a critical defense mechanism to protect the body from oxidative stress in nervous system disorders (Bartolini et al., 2018). Researchers have suggested that the P62 protein may link autophagy to Nrf2 and facilitate intercommunication *via* the autophagy-lysosome pathway. In only a few days following trauma, oxidation species over-express in tissue, damaging proteins, lipids, and DNA structure (Deshmukh et al., 2017). In addition, the membrane permeability and integrity are compromised due to the depletion of endogenous antioxidants. Keap1 and Nrf2 complex formation is necessary under normal conditions, however, P62 protein can compete with Nrf2 for the Kelch binding site of Keap1 when P62 protein accumulates heavily after autophagic flux blockade. When this occurs, Nrf2 immediately transfers from the cytoplasm to the nucleus to activate transcription against oxidative damage. Lv et al. demonstrated that polydatin acts on Nrf2/HO-1 pathway to protect spinal cord microglia (Lv et al., 2019). *In vitro* studies demonstrated that the addition of aucubin promotes the gradual transfer of Nrf2 to the nucleus and reduces the levels of reactive oxygen species, while *in vivo* studies showed that the antioxidant effect of aucubin is mediated by the Nrf2 pathway using the Nrf2 knockout models before and after treatment (Wang et al., 2020; Figure 2B).

Beclin1 pathway (Tran et al., 2021), nuclear factor kappa-light-chain-enhancer of activated B cells (NF-κB) pathway (Zhou et al., 2018) and others are additional signaling pathways involved in the regulation of autophagy. Notably, the AMPK and the mTOR pathways regulate metabolic homeostasis as two opposites of yin and yang (González et al., 2020). The complex cross-links between the signal pathways and the hidden links between the pathways must be continuously studied.

The upstream molecules mediate the activation of autophagy, including mTOR, AMPK, PI3K/AKT, and Nrf2. In addition, activate forkhead box O 3a (FoxO3a), which is strongly associated with glucose metabolism (Liu Z. et al., 2015), energy-related dynamin-related protein 1 (Drp1) (Song et al., 2015), and toll-like receptor 4 (TLR4)/nuclear factor kappa-light-chain-enhancer of activated B cells (TLR4/NF-κB) pathway relevant to inflammation also regulate the progress of autophagy (Fang et al., 2013; Zhang and Wang, 2018). The downstream of autophagy receives signals and provides neuroprotection by increasing cognitive ability (Zhang et al., 2021), inhibiting oxidative stress (Cui et al., 2021), apoptosis (Chen et al., 2018), and inflammation (Levine et al., 2011).

## 3. The interaction of autophagy with apoptosis, inflammation and angiogenesis in CNS trauma

### 3.1. Autophagy crosstalk apoptosis in CNS trauma

As we all know, apoptosis, autophagy cell death and necrosis occur after injury. Both autophagy and apoptosis accompany trauma and closely regulated biochemical mechanisms between them to determine the life of the cell, the Bcl-2 family is particularly important at this time (Mariño et al., 2014). Anti-apoptotic proteins-Bcl-xl and Bcl-2-jointly regulate autophagy and apoptosis, BH3 pro-apoptotic structure can accelerate the separation of Bcl-2 and Bcl-xl proteins from Beclin1 through competitive binding. With AMPK, Beclin1-PI3K complex formation is promoted to enhance autophagy and maintain low level of Bcl-2, and Bcl-xl proteins, promoting neuronal survival in the injured area after CNS injury (Zhao et al., 2022). Qin et al. (2022) found from experiment that long non-coding RNA LINC00158 overexpression repressed neuronal apoptosis by inducing autophagy, while 3-Methyladenine and chloroquine deteriorate this phenomenon, as a result of increased apoptosis in spinal cord tissue. Poly-arginine R18 reduced neurocyte apoptosis and promoted neurocyte cell growth in TBI model (Batulu et al., 2019). However, autophagy and apoptosis are not simply cooperative, also antagonistic. Fibroblast growth factor-2 (FGF2) protected cells from various forms of death such as apoptosis or necrosis to play a neuroprotective role through inhibition of autophagy, and got an opposite effect after intervention with rapamycin in mild TBI (Tang et al., 2017). After brain trauma, there is a phenomenon characterized by significantly increased levels of LC3-II biomarkers within 48 h. Pretreatment with 3-MA decreased the overall rise in autophagy activation-induced LC3 and lowered the expression of cathepsins B, which may contribute to cell death. Consequently, autophagy may interact with cell death (Luo et al., 2011). Therefore, the complex interaction between autophagy and apoptosis remains a major challenge for current research.

### 3.2. Autophagy modulates inflammation in CNS trauma

The inflammatory response is present throughout the development of CNS trauma. After injury, astrocytes and microglia are activated to varying degrees, releasing a variety of inflammatory factors such as TNF-α, IL-1β, and IL-6, which amplify the neuroinflammatory response and extend the normal tissue to be damaged accordingly. Neural stem cell-derived small extracellular vesicles treatment can regulate apoptosis and inflammatory processes by inducing autophagy to promote functional recovery in damage model of spinal cord (Rong et al., 2019). Conversely, inhibition of autophagy after SCI potentiates pro-inflammatory activation in microglia, and complementary evidence through transcriptomics, cellular, and molecular studies provided ample evidence that defective autophagy exacerbates neuroinflammation and is associated with worse functional outcomes following insult (Li et al., 2022). Stimulator of interferon genes (STING) often perceived as key mediators of the inflammatory reaction in the central nervous systems, they concluded a detrimental role for STING in mediating the TBI-induced neuroinflammatory response and autophagy dysfunction (Abdullah et al., 2018). All of above findings can be confirmed that inhibition of inflammatory process acts as a useful tool for CNS trauma *via* the modulation of autophagy.

### 3.3. Autophagy modulates angiogenesis in CNS trauma

The regulation of angiogenesis by autophagy is dual in nature. Autophagy-related proteins and inflammatory factors can promote angiogenesis by regulating cellular autophagy after spinal cord or brain injury. Knockdown of autophagy-related proteins can inhibit angiogenesis. Wan and co-authors verified that G protein-coupled receptor kinase 2 interacting protein-1 (GIT1) may prompt microvascular endothelial cells to clear myelin debris *via* autophagy and further stimulate microvascular endothelial cells angiogenesis *via* upregulating VEGF (Wan et al., 2021). NADPH oxidase 2 (NOX2)-induced ROS production is a double-edged sword that exacerbated brain injury in the acute phase but promoted functional recovery, this effect appears to be achieved by inhibiting NLRP3 inflammasome activation and promoting angiogenesis *via* PI3K/Akt autophagy pathway activation (Yingze et al., 2022). Endothelial cells exerted critical functions to promote inflammation and angiogenesis and may contribute to fibrotic scar formation by autophagy-lysosome pathway in both SCI and EAE after neural injury (Zhou et al., 2019). RTN4-S1PR2 suppressed angiogenesis in the ipsilateral thalamus by bafilomycin A1 treatment, conversely, inhibition of autophagy initiation with 3-methyladenine enhanced angiogenesis. This means that RTN4-S1PR2 negatively regulates angiogenesis through enhancing autophagy (Xiao et al., 2022). Moreover, some researchers found that the inhibition of angiogenesis was reduced when ATG-7 was knocked out or inhibited using 3-MA (Codogno et al., 2011; Alers et al., 2012).

## 4. The role of autophagy in CNS trauma

Multiple studies have been carried out on the action between autophagy and central nervous damage, however, the function and role are not yet fully understood. Kanno et al. (2009) first described the interaction between the spinal cord and autophagy through the expression of Beclin1 in hemisection models of mice spinal cord. Erlich et al. (2007) hypothesized that autophagy plays a protective role in adult mice TBI models treated with rapamycin. Autophagy plays a key and complex role in the study of CNS through recovering and degrading damaged organelles and misfolded proteins and has become a hot topic. However, it has been controversial whether it plays a protective or a detrimental role (Lipinski et al., 2015). The level of autophagy flux and the number of autophagosomes correlate closely with autophagy activity (Zhou et al., 2017; Nakatogawa, 2020).

### 4.1. Possible neurological protective role of autophagy

Upregulation of autophagy levels is considered a potential therapeutic strategy as damaged organelles and proteins accumulate in neurons after injury has occurred (Gu et al., 2021). Animal models such as mice and rats can represent human brain or spinal cord damage. Recent studies have reported that autophagy flux trends vary between models (Ray, 2020). In traumatic injuries such as spinal cord contusion model, Beclin1 and LC3-II reached their peak 2 h after injury and enhanced Basso-Beattie-Bresnahan scale (BBB) in rats, indicating that the autophagy level was improved, and functional recovery was accelerated after injury (Hao et al., 2013). During hemisection injury, however, LC3 radioactivity increased significantly and peaked at 3 days, as determined by immunohistochemical analysis. In the non-traumatic rat model involving spinal cord ischemia and reperfusion injury rat model, Fang et al. (2016) and Xiong et al. (2020) found that autophagy flux was induced by blocking the aorta for 60 and 14 min, respectively. However, the specific temporal pattern requires further investigation.

Increasing evidence indicates that reactive oxygen species (ROS) levels can modulate autophagy flux. Following SCI, damaged tissue generates redundant reactive oxygen species, resulting in an uneven distribution of ROS and activation of the oxidative stress system (Salim, 2017). ATG4 is a molecule that detects ROS in the body or cell and can enhance the lipidation process of LC3 to enable autophagy directly when ROS levels are elevated. H_2_O_2_ is one of the forms of ROS that can indirectly activate autophagy by modifying the AMPK subunit to upregulate AMPK activity (Zmijewski et al., 2010). Tong et al. (2018) studied the role of lithium chloride (LiCl) in autophagy and SCI using Western blotting in rat animal models. The experiments showed that LiCl upregulated ATG5 and LC3-II expression and downregulated the expression of P62, indicating its protective role in damaging the spinal cord and neural cells by inducing autophagy flux (Tong et al., 2018). In the SCI model developed by Bisicchia et al. (2017) it was observed that LC3-positive autophagosomes accumulated in neurons while the expression of LC3II/LC3I and p62 increased, indicating that the decrease in autophagosome clearance caused this accumulation, autophagy inhibitor 3-MA could reduce autophagosome formation, reduce degeneration, and improve spontaneous functional recovery. Activation of autophagy reduces damage to cortical neurons and provides a certain degree of protection against moderate brain injury by inhibiting the formation of inflammatory vesicles (Gao et al., 2020c).

### 4.2. Possible neurological damage role of autophagy

Autophagy flux is activated after mild or moderate injury and inhibited after severe injury. Excess reactive oxygen species and pro-apoptotic proteins are produced in the body when severe SCI occurs, which inhibit autophagy *via* the mTOR pathway and result in neuronal death (Yun et al., 2020). In addition, autophagy degradation is lysosome-dependent, thus lysosomal dysfunction, as a negative regulator factor for autophagy flux, causes autophagosome accumulation (Ballabio and Bonifacino, 2020). Liu S. et al. (2015) observed that the decrease in lysosomal protease cathepsin D was accompanied by an increase in LC3-II and P62 in SCI rats, indicating that autophagy was induced, but degradation was inhibited, ensuing aggravation of the injury. Neuronal apoptosis and worsened neurological recovery are reported to have extensive communication with impaired autophagy (Yin et al., 2017). Sarkar et al. (2014) found that P62 was increased in both the cortex and hippocampus of brain-injured rats, suggesting that inactive lysosomal inhibits autophagy and negatively regulates neurological function. Other data revealed a high neuronal survival rate and fewer nervous system defects in VPS34 gene-deficient rats compared to normal rats, indicating that nociceptors are also protected by a reduction in excess autophagosomes (Quan et al., 2021).

**FIGURE 3:**
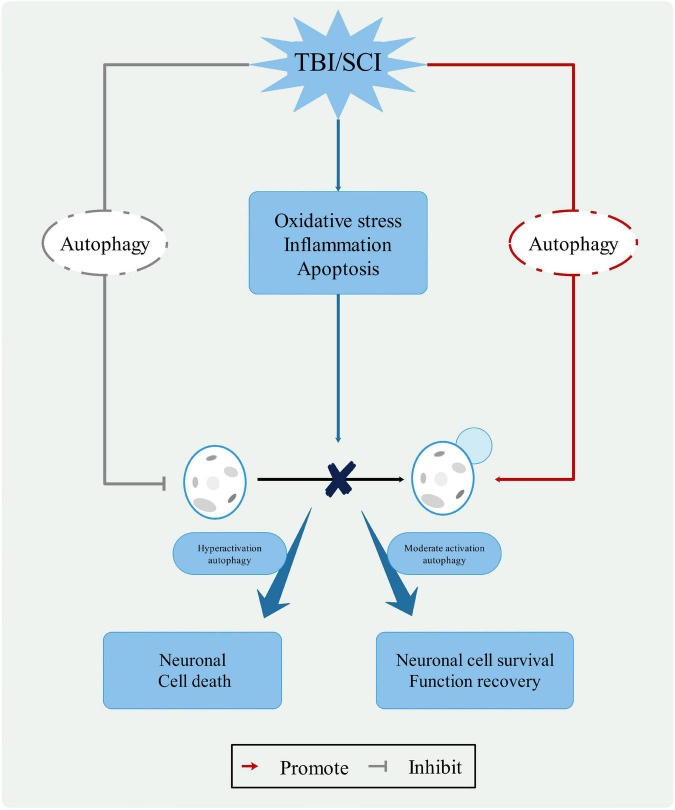
The role of autophagy in CNS trauma. When the CNS is damaged, changes in the pathophysiological processes, such as oxidative stress, inflammatory response, and apoptosis, leading to changes in the lysosomal membrane and blockage of the autophagy process. Moderate activation autophagy can protect neuronal cell and function recovery, hyperactivation autophagy causes neuronal cell death. Therefore, autophagy pathway represents a potential clinical therapeutic value for treatment of CNS trauma.

Autophagy, as a “Trash Disposal Center,” plays a dual role in the neurological function of CNS injuries (Kabadi and Faden, 2014; Ray, 2020). By stimulating autophagy flux and inducing neuronal apoptosis, neuronal cells are protected through the acceleration of autophagy degradation. The antithesis is the inhibition of autophagic flux and autophagy overload, which exacerbates neurological lesions (Wu and Lipinski, 2019). The level of autophagy is a function of both the environment and the severity of the trauma. Mild injury has almost no effect on autophagy, moderate injury protects damaged neurons, and severe injury induces apoptosis. Notably, the temporary inhibition of autophagic flux following injury may have the opposite effect when autophagic flux is activated (Zmijewski et al., 2010). Therefore, further research is needed to understand the role of autophagy (Figure 3).

## 5. Targeting autophagy therapeutics in CNS trauma

The likelihood of a permanent cure for diseases of the central nervous system is insufficiently high. Currently, surgical and pharmacological interventions are the most prevalent treatment options for CNS diseases. Numerous studies indicate that some drugs can mediate autophagy and play an effective therapeutic role in CNS injury. Hence, we mainly looked for drugs that protect neurons by inducing or promoting autophagy can be divided into two main categories: drugs that protect neurons by inhibiting or blocking autophagy in the last 5 years (Table 1).

**TABLE 1:** Autophagy-targeted therapeutic agents for neuroprotection and functional recovery in central nervous trauma.

Agent (s)	Regulation of autophagy	Mechanisms	Action pathway	References
Rapamycin	Induction	Activate of autophagy flux, inhibit mTOR activation, decrease inflammation, reduce neuronal apoptosis	No mention	Sekiguchi et al. (2012), Chen et al. (2013), Goldshmit et al. (2015)
Betulinic acid	Induction	Restore autophagy flux, eliminate the accumulation of ROS	AMPK-mTOR-TFEB	Wu et al. (2021)
Salidroside	Induction	Stimulate autophagy flux, reduce edema and apoptosis	AMPK/mTOR PI3K/Akt	Chen et al. (2012), Wang C. et al. (2018)
Eugenol	Induction	Enhance autophagy, alleviate inflammation, oxidative stress, and neural apoptosis	AMPK-mTOR-P70S6K NF-κB	Ma et al. (2018), Sun et al. (2020)
Resveratrol	Induction	Activate neuronal autophagy, inhibit neuroinflammation and activate oxidative stress	AMPK/mTOR NF-κB, Nrf2	Kesherwani et al. (2013), Meng et al. (2018), Wang P. et al. (2018), Xu L. et al. (2018), Pineda-Ramírez et al. (2020)
Anthocyanins	Induction	Increase autophagy and reduce apoptosis	SIRT1/AMPK PI3K/AKT/mTOR	Ding et al. (2015), Li et al. (2019), Gao et al. (2020a)
Curcumin	Induction	Reduce neuron apoptosis, improve spinal cord integrity, recovery, and re-myelination, and suppress the inflammatory response	Akt/mTOR	Forouzanfar et al. (2020), Li et al. (2021)
Chloroquine	Inhibition	Inhibit autophagy-associated inflammation and endoplasmic reticulum stress	No mention	Wu et al. (2018), Xu R. et al. (2018)
3-MA	Inhibition	Inhibit of autophagosomes formation, attenuate remote degeneration and improve spontaneous functional recovery	No mention	Bisicchia et al. (2017)
Hydrogen sulfide	Inhibition	Improve memory function, Inhibiting Endoplasmic Reticulum Stress-Dependent Autophagy	No mention	Karimi et al. (2017), Wang H. et al. (2018)
Ketamine	Inhibition	Exert anti-inflammatory, decrease autophagy-associated protein synthesis	mTOR	Wang et al. (2017)
Stilbene glycoside	Induction Inhibition	Attenuate injury, improving learning, memorizing, and spatial orientation behavior	AMPK/PINK1/parkin	Luo et al. (2015), Gao et al. (2020d)
Wnt3a	Induction Inhibition	Significantly reduced the loss of neurons, reduced cell death, promote neuroprotection and neural regeneration	mTOR Wnt/β-catenin	Zhang et al. (2018), Gao et al. (2020b)

### 5.1. Drugs that induce or promote autophagy in CNS trauma

Rapamycin, as a non-nephrotoxic antibacterial drug and autophagy activator, has also been shown to contain anti-inflammatory properties. It targets mTORC1 action (Li et al., 2014). Goldshmit et al. (2015) believed that rapamycin is most probably useful in acute SCI *via* injecting it once into neurons and astrocytes in the cell, which inhibits p62 expression and proliferation of astrocytes, thereby reducing inflammatory factor invasion at the damaged site. Sekiguchi et al. (2012) and others concluded that rapamycin benefits mice with TBI and SCI (Chen et al., 2013; Gao et al., 2020c). Transcription factor EB (TFEB), acting as the primary regulator of the autophagy-lysosome pathway (Cortes and La Spada, 2019), was involved in regulating autophagy. Betulinic acid is largely extracted from the bark and leaves of birch trees. It acts on the AMPK-mTOR-TFEB signaling pathway to inhibit spinal proptosis by increasing autophagy and decreasing ROS levels (Wu et al., 2021). Salidroside is a well-known extract of the Chinese herb Rhodiola, stimulating autophagy flux through AMPK/mTOR exerts anti-apoptotic levels of Salidroside in neuronal cells near the site of SCI (Wang C. et al., 2018). In addition, PI3K/Akt signaling can partially downregulate edema and apoptosis following TBI (Chen et al., 2012). Eugenol, resveratrol, anthocyanins and curcumin are phenolic compounds. They interact with receptors or enzymes at the site of injury to modulate the activity of signaling pathways involved in improving disease and cognitive function. Eugenol is one of the active components of the traditional Chinese medicine Acorus Tatarinowii Schott, which protects the brain from cerebral ischemia-reperfusion injury. *In vivo* and *in vitro* studies have shown that eugenol can mitigate brain injury by modulating the AMPK/mTOR/P70S6K signaling pathway (Sun et al., 2020). In addition, eugenol affects SCI by inhibiting oxidative stress and inflammation systems in the damaged spinal cord (Ma et al., 2018). Previous studies have demonstrated that resveratrol improves experimental SCI or TBI models by inhibiting inflammatory signaling pathways and activating oxidative stress (Kesherwani et al., 2013; Xu L. et al., 2018). In addition, resveratrol influences the autophagy process by activating the AMPK/mTOR signaling pathway, intending to enhance the protection of damaged cells and neurological function (Meng et al., 2018; Pineda-Ramírez et al., 2020). Anthocyanins, as a natural pigment, protect against both spinal cord and brain injuries by regulating autophagy. Apigenin, one of the anthocyanins in flavonoids, is primarily found in temperate or tropical zone vegetables, fruits and tea, with celery having the highest content. Kim et al. (2011) found that apigenin inhibited the progression of neurodegenerative lesions. Melatonin can regulate the immune system, eliminate free radicals, and has neuroprotective properties. Melatonin can act on SIRT1/AMPK or PI3K/AKT/mTOR, two pathways to increase autophagy and reduce apoptosis (Ding et al., 2015; Li et al., 2019; Gao et al., 2020a). Curcumin derived from *Curcuma longa* exhibits anti-cancer and antioxidant effects in clinical settings. It can reduce the brain content and improve cell death following brain injury (Forouzanfar et al., 2020). Also promotes recovery of hind limb function after SCI (Li et al., 2021).

### 5.2. Drugs that inhibit or block autophagy in CNS trauma

Chloroquine (CQ) is a common autophagy inhibitor that disrupts the integration process of autophagosomes with lysosomes to protect the organism (Wu et al., 2018; Xu R. et al., 2018), 3-MA widely used as an inhibitor of autophagy by inhibiting class III PI3K (Bisicchia et al., 2017), both of them significantly blocked the formation of autophagy to promoting neuroprotection in trauma regions. Hydrogen sulfide (H_2_S) is reportedly a new protective gas for neurological function, with NaHS as its donor. Indeed, inhibition of autophagy and improved memory scores were observed with NaHS treatment after TBI (Karimi et al., 2017). After combined treatment with NASH and autophagy activator compared to only NASH in SCI, negative improvement in blood-spinal cord barrier (BSCB) permeability was observed (Wang H. et al., 2018). Ketamine is an intravenous anesthetic, and its neuroprotective effects have recently been studied from a new angle. Experiments showed that ketamine affects the activity of the mTOR pathway and promotes its suppression of autophagy to improve injury recovery (Wang et al., 2017).

In addition, it is concluded that specific drugs have dual effects on the regulation of autophagy, which can improve cerebral injury by inducing autophagy and protect neurons from excessive injury by inhibiting autophagy. Stilbene glycoside, a kind of monomer component with a neuroprotective effect, is isolated from *Fallopia multiflora*. It has numerous pharmacological effects, including antioxidant, hypolipidemic, anti-inflammatory, detoxification, and neuroprotective properties. Studies have shown that stilbene glycosides can exert a role in improving behavioral disorders effect in a mouse model by inhibiting (Luo et al., 2015) or promoting autophagy (Gao et al., 2020d). The regulatory effect of Wnt3a on autophagy also belongs to this category, which could activate in the SCI model by inhibiting the mTOR pathway and inhibit in TBI (Zhang et al., 2018; Gao et al., 2020b).

Currently, there are feasibility studies for drugs used to treat one of two injuries, which can serve as a benchmark for the other. In addition, the same compound can modulate autophagy in injured tissues by acting on different regulatory signaling pathways. In short, target studies of autophagy are a new and viable gateway to the development of CNS trauma. Although there are relatively few clinical trials of these agents at present, their discovery opens up more and more possibilities for repairing central nervous damage, prolonging the survival period and enhancing the well-being of patients after injury.

## 6. Conclusion

Despite rapid medical advancements over the past two decades, brain and spinal cord trauma remains one of humanity’s greatest challenges. Mild or moderate autophagy is considered as an endogenous mechanism for protecting neurons, whereas excessive autophagy exacerbates neuronal injury. Much interest has been in modulating autophagy as a new therapeutic modality for CNS injury. Numerous compounds have been investigated to modulate autophagy by activating, promoting or inhibiting autophagy through various signaling pathways or targets to improve neural injury and increase cell survival duration. However, it appears that autophagy exerts beneficial effects within a certain interval, but how this interval is quantified and whether the regulatory effect on autophagy in previous studies is to adjust autophagy levels to this beneficial interval remains to be further investigated. In addition, there is a problem with the current research on autophagy, which is the monitoring of autophagy levels in injured patients. If autophagy levels could be controlled by detecting metabolites secreted *via* autophagy-dependent pathways in patients, “saving” yourself so future research is necessary. Targeting the autophagy process for the treatment of CNS injury promises to provide more opportunities and directions for the future development of therapeutic drugs.

## Author contributions

SW had written the original draft. GY revised and edited the manuscript. BL designed and provide the graphic image. All authors have read and agreed to the published version of the manuscript.
