# Corrigendum: Targeting autophagy process in center nervous trauma

**DOI:** 10.3389/fnins.2023.1201138

**Published:** 2023-05-11

**Authors:** Shanshan Wei, Bing Leng, Genquan Yan

**Affiliations:** ^1^Department of Pharmacy, Shandong Provincial Hospital Affiliated to Shandong First Medical University, Jinan, China; ^2^Department of Graduate, Shandong Academy of Medical Sciences, Shandong First Medical University, Jinan, China

**Keywords:** autophagy, central nervous trauma, spinal cord injury (SCI), traumatic brain injury (TBI), autophagy flux

In the published article, there was an error in affiliation(s) [1, 2]. Instead of “Shanshan Wei^1, 2^, Bing Leng^2^ and Genquan Yan^2*^, ^1^Department of Graduate, Shandong Academy of Medical Sciences, Shandong First Medical University, Jinan, China, ^2^Department of Pharmacy, Shandong Provincial Hospital Affiliated to Shandong First Medical University, Jinan, China,” it should be “Shanshan Wei^1, 2^, Bing Leng^1^ and Genquan Yan^1*^, ^1^Department of Pharmacy, Shandong Provincial Hospital Affiliated to Shandong First Medical University, Jinan, China, ^2^Department of Graduate, Shandong Academy of Medical Sciences, Shandong First Medical University, Jinan, China.”

The authors apologize for this error and state that this does not change the scientific conclusions of the article in any way. The original article has been updated.

